# Synthesis, Characterization, and Application Prospects of Novel Soluble Polysilsesquioxane Bearing Glutarimide Side-Chain Groups

**DOI:** 10.3390/polym16233235

**Published:** 2024-11-21

**Authors:** Yuliya I. Bolgova, Artem I. Emel’yanov, Olga M. Trofimova, Anastasiya A. Ivanova, Alexander I. Albanov, Nadezhda P. Kuznetsova, Tatyana A. Semenova, Alexander S. Pozdnyakov

**Affiliations:** A.E. Favorsky Irkutsk Institute of Chemistry of the Siberian Branch of the Russian Academy of Sciences, 1 Favorsky Str., 664033 Irkutsk, Russia; omtrof1@irioch.irk.ru (Y.I.B.); emelyanov@irioch.irk.ru (A.I.E.); omtrof@irioch.irk.ru (O.M.T.); ivanova93@irioch.irk.ru (A.A.I.); albanov@irioch.irk.ru (A.I.A.); nkuznetsova@irioch.irk.ru (N.P.K.); semjenova@irioch.irk.ru (T.A.S.)

**Keywords:** silsesquioxane, glutarimide, trimethoxy(propyl)silane, hydrolytic polycondensation, film coating

## Abstract

The requirement for the development of advanced technologies is the need to create new functional thermostable soluble polysilsesquioxanes. Combining the potential of organosilicon chemistry and the chemistry of heterocyclic compounds is a promising direction for the formation of novel organosilicon polymer systems with new properties and new possibilities for their practical application. Using the classical method of hydrolysis and polycondensation of previously unknown trifunctional (trimethoxysilylpropyl)glutarimide in the presence or absence of an acid or base catalyst, a universal approach to the formation of new thermostable soluble polysilsesquioxanes with glutarimide side-chain groups is proposed, which forms the basis for the synthesis of polysilsesquioxane polymers with different functionality. The weight average molecular weight of silsesquioxanes, determined by gel permeation chromatography, is practically independent of the reaction conditions and is 10–12 kDa; at the same time, the molecular weight distribution remains low and amounts to 1.38–1.47. According to thermogravimetric analysis, the resulting polysiloxanes have high thermal stability up to 335 °C. By the dynamic light scattering method, it was established that in an aqueous solution, silsesquioxane macromolecules are in an associated state, forming supramolecular structures due to the intermolecular interaction of individual macromolecules. The average hydrodynamic diameter of the particles was 46 nm. X-ray diffraction analysis showed the amorphous nature of the polymer. Polymer film coatings based on synthesized silsesquioxanes are characterized by 98% transmission in the visible spectrum and resistance to ultraviolet radiation, which is promising for the creation of functional transparent film coatings.

## 1. Introduction

Silsesquioxanes are organo-inorganic hybrids with a general chemical formula of (RSiO_1,5_)_n_, which have a stable inorganic silica-like framework surrounded by organic substituents attached to the silicon atom. The main advantage of silsesquioxane structures is the variety of peripheral organic groups providing functionalization, which leads to a wide range of properties and, therefore, to various applications of these molecules. Silsesquioxanes are a universal class of three-dimensional organosilicon compounds that have been the subject of intensive research due to their combination of practically important and unique properties for a long time. These are high chemical, thermal, and mechanical stability, hydrophobicity, low dielectric constant, and biocompatibility [1,2,3,4]. Interest in silsesquioxanes continues to grow steadily due to their widespread use as protective coatings, insulating layers, aerospace materials, catalyst carriers, ceramic precursors, photoresistors, etc. [5,6,7].

Available literature data show that polysilsesquioxanes are UV-resistant materials due to the presence of inorganic Si–O bonds in them [8,9,10,11,12,13]. UV-resistant materials have attracted much attention due to their important practical applications. It is known that polymer coatings gradually degrade when used outdoors due to exposure to ultraviolet radiation, oxygen, moisture, and other factors. UV radiation leads to the destruction of the polymer chain and loss of strength at a number of points in the structure. One of the ways to protect materials from destructive ultraviolet radiation is to use polymer coatings based on polymers containing chromophore groups, such as carbonyl, carboxyl, or nitrile, which have absorption in the ultraviolet spectrum. Of particular interest from the point of view of obtaining protective polymer coatings are polysilsesquioxanes with organic substituents in the side chains, possessing the properties of a UV absorber that does not change the transparency of the polymer in the visible region of the spectrum and having high photostability and absorption capacity in the UV region of the spectrum. Therefore, the synthesis of novel silsesquioxanes and the development of transparent UV-protective coatings based on them are considered necessary and in demand for the creation of materials that can protect against ultraviolet radiation. The properties of materials are determined to a greater extent by the chemical structure of the inorganic core, as well as the possibility of varying the nature and number of functional groups in organic substituents associated with silicon atoms. Many nitrogen-containing heterocyclic compounds have physiological and pharmacological properties and are components of biologically important molecules such as vitamins, nucleic acids, antibiotics, pharmaceuticals, dyes, agrochemicals, and others [14,15,16]. They have found wide application as synthons of organic synthesis for creation of new molecules and composites, as well as in medicinal chemistry, pharmaceutical industry, and agriculture [17,18,19,20,21,22]. In this regard, at present, a promising direction for the creation of new functionalized organosilicon polymer systems with new properties and new practical application possibilities is to combine the potential of chemistry of organosilicon and nitrogen-containing heterocyclic compounds.

Previously, we synthesized functional polysilsesquioxane with phthalimide side-chain groups [23]. Continuing these researches, we became interested in the synthesis, study of the physicochemical characteristics, and search for practical applications of other cyclic imides of dicarboxylic acids. In this study, we present the synthesis of a novel representative of the class of thermostable soluble polysilsesquioxanes with glutarimide side-chain groups using a simple and convenient classical method of hydrolytic polycondensation. Glutarimide (2,6-piperidinedione), which belongs to chromophores (λ_max_ 204 nm) containing two carbonyl groups, is a component of a number of molecules with a wide range of biochemical and pharmacological activities [24,25,26,27]. The synthesis, structure, and properties of a previously unknown monomer 1-[3-(trimethoxysilyl)propyl]piperidine-2,6-dione and functional polymers based on it are discussed. Polymer coatings based on the synthesized silsesquioxanes demonstrated high transmittance in the visible spectrum and resistance to ultraviolet radiation, which makes them promising for the creation of functional transparent protective film coatings.

## 2. Materials and Methods

### 2.1. Materials

Piperidine-2,6-dione (glutarimide, GI) (98%), sodium hydroxide (NaOH, ≥98%, pellets (anhydrous)), and hydrochloric acid (HCl, ACS, 37%) were purchased from Sigma-Aldrich (Munich, Germany) and used without further purification. The sodium salt of piperidine-2,6-dione is synthesized by the reaction of sodium methoxide and glutarimide. Piperidine-2,6-dione sodium salt was prepared by the reaction of sodium methoxide with glutarimide. Sodium methoxide was prepared by carefully adding freshly cut metallic sodium (1.13 g, 49.2 mmol) to an excess of anhydrous methanol (7.88 g, 246.0 mmol) immediately before use. To remove moisture, methanol (CH_3_OH, ACS, ≥99.8%, Sigma-Aldrich, Munich, Germany) was kept over magnesium for 12 h and then distilled. The initial (3-chloropropyl)trimethoxysilane was obtained by following the method of the literature [28] by alkoxylation of (3-chloropropyl)trichlorosilane. (3-Chloropropyl)trichlorosilane was produced according to a literature method [29]. *N*,*N*-Dimethylformamide (DMF, 99.8%, Sigma-Aldrich, Munich, Germany) was distilled with calcium hydride immediately before use to remove moisture. Deionized water (resistivity ≥17.5 MΩ∙cm, Vodoley-M water purifier, RU) was used for all the aqueous solutions and in the preparation of silsesquioxane.

### 2.2. Synthetic Procedures

#### 2.2.1. Synthesis of 1-[3-(Trimethoxysilyl)propyl]piperidine-2,6-dione monomer **1**

To a solution of piperidine-2,6-dione sodium salt (7.53 g, 49.2 mmol) in DMF (30 mL), in the presence of dibenzo-24-crown-8 ether (0.018 g, 0.04 mmol), (3-chloropropyl)trimethoxysilane (9.78 g, 49.2 mmol) was added dropwise. The reaction mixture was stirred at 70 °C for 2 h, and the resulting sodium chloride precipitate was filtered off. The filtrate was then distilled under reduced pressure, yielding 12.19 g of target monomer **1** with a yield of 90%. The boiling point of the product was 166 °C (2.00 torr), and its melting point was 42 °C. Anal. Calc. (%) for C_11_H_21_NO_5_Si: C 47.98, H 7.69, N 5.09, Si 10.20. Found (%): C 47.62, H 7.71, N 4.73, Si 9.82. FTIR (ν, cm^−1^): 2946 (CH_2_), 2842 (C–H in Me), 1724, 1673 (C=O), 1460–1433, 1387–1355 (C–C, piperidine-2,6-dione cycle), 1312 (C–N), 1192 (Si–OMe), 1084 (Si–O), 1016 (C–O), 816 (Si–OMe). ^1^H NMR spectrum (400 MHz, CDCl_3_, δH, ppm): 3.74–3.71 (m, 2H, NCH_2_), 3.54 (s, 9H, OCH_3_), 2.62 (t, 4H, ^3^*J* = 6.4 Hz, CH_2_C(O), piperidine-2,6-dione ring protons), 1.91 (qu, 2H, ^3^*J* = 6.4 Hz, CH_2_C*H*_2_CH_2_, piperidine-2,6-dione ring protons), 1.63–1.55 (m, 2H, CH_2_C*H*_2_CH_2_Si), 0.59–0.63 (m, 2H, CH_2_Si). ^13^C NMR spectrum (100 MHz, CDCl_3_, δC, ppm): 172.49 (C=O), 50.65 (OCH_3_), 42.07 (NCH_2_), 33.01 (*C*H_2_C(O), piperidine-2,6-dione ring), 21.35 (CH_2_*C*H_2_CH_2_Si), 17.32 (CH_2_*C*H_2_CH_2_, piperidine-2,6-dione ring), 6.70 (CH_2_Si). ^29^Si NMR spectrum (79.5 MHz, CDCl_3_, δSi, ppm): −42.4. MS *m*/*z* (*I*_rel_, %): 275 [M]^+^ (0.09), 274 [M–H]^+^ (0.11), 244 [M–OMe]^+^ (10), 243 [M–OMe–H]^+^ (45), 202 [M–(CH_2_)_3_–OMe]^+^ (66), 162 [M–(CH_2_)_3_(C=O)_2_N–H]^+^ (5), 121 [Si(OMe)_3_]^+^ (100), 91 [Si(OMe)_3_–OMe+H]^+^ (40), 90 [Si(OMe)_3_–OMe]^+^ (6), 77 [Si(OMe)_3_–Me_3_+H]^+^ (6), 61 [Si(OMe)_3_–OMe–Me_2_+H]^+^ (13), 59 [Si(OMe)_3_–(OMe)_2_]^+^ (12).

#### 2.2.2. Synthesis of 1-[3-(Silsesquioxanyl)propyl]piperidine-2,6-dione (PSQ-GI) **2a**–**c**

The monomer 1-[(3-trimethoxysilyl)propyl]piperidine-2,6-dione **1** was polymerized under solvent and catalyst-free conditions using deionized water (pH 7). Deionized water, which had been preliminary adjusted to have a pH of 3.0 or 10.0, was used as well. Deionized water (2 mL) was added to the monomer **1** (0.275 g, 1.0 mmol) in a single drop with stirring. After the addition, the reaction mixture became clear and homogeneous. The reaction mixture was then heated to 70 °C and maintained at this temperature for 8 h with vigorous stirring. After cooling to room temperature, a water–alcohol phase was separated using a rotary evaporator. If hydrochloric acid or sodium hydroxide catalysts were used, the following additional steps were taken to neutralize the reaction mixture: the mixture, after separating the water alcohol layer, was washed with deionized water to achieve a pH of 7 in the eluate. The remaining clear viscous substance was dried in a vacuum at room temperature for 24 h. The final polysilsesquioxanes **2a**–**c** containing glutarimide side-chain groups were obtained as colorless solids. The yield of silsesquioxanes **2a**–**c** was 78–87%, based on the ideal chemical formula for one unit of this product, [C_8_H_12_NSiO_3.5_, FW = 206.277]. Anal. Calc. (%) for C_8_H_12_NO_3.5_Si: C 46.58, H 5.86, N 6.79, Si 13.62. Found (%): C 46.02, H 5.90, N 6.28, Si 14.17. FTIR (ν, cm^−1^): 3376 (O–H), 2955, 2885 (CH_2_), 1722 and 1662 (C=O), 1457–1435, 1384–1353 (C–C, piperidine-2,6-dione ring), 1120 and 1042 (Si–O–Si). ^1^H NMR spectrum (400 MHz, CDCl_3_, δH, ppm): 3.65 (br, 2H, NCH_2_), 2.59 (br, 4H, CH_2_C(O), protons of the piperidine-2,6-dione ring), 1.89 (br, 2H, CH_2_C*H*_2_CH_2_, protons of the piperidine-2,6-dione ring), 1.49 (br, 2H, CH_2_C*H*_2_CH_2_Si), 0.53 (br, 2H, CH_2_Si). ^13^C NMR spectrum (100 MHz, CDCl_3_, δC, ppm): 172.54 (C=O), 41.79 (NCH_2_), 32.86 (*C*H_2_C(O), the piperidine-2,6-dione ring), 21.23 (CH_2_*C*H_2_CH_2_Si), 17.19 (CH_2_*C*H_2_CH_2_, the piperidine-2,6-dione ring), 9.78 (*C*H_2_Si). ^29^Si NMR spectrum (79.5 MHz, CDCl_3_, δSi, ppm, chemical shifts were assigned according to the literature [5,30,31]): −53.5 to −59.4 (T^2^) and −62.7 to −69.1 (T^3^).

#### 2.2.3. Preparation of a Polymer Film Coating Based on PSQ-GI

To prepare a polymer film coating based on 1-[3-(silsesquioxanyl)propyl]piperidine-2,6-dione, 104.0 mg of polysilsesquioxane was dissolved in 0.70 mL of DMSO. The resulting homogeneous solution was then filtered through a 0.45 μm PTFE syringe filter. Then, the solution was divided into equal parts and applied to the silicate and quartz glass plates by casting. To improve the adhesion of the coating, the substrates were pre-treated with detergent and acetone for 15 min. After each treatment, they were thoroughly washed with distilled water and dried at 100 °C for 30 min. The film samples were air-dried at room temperature for two days to slowly evaporate the bulk of the solvent, then heated at 50 °C for 2 h and further dried at 60 °C at 2 mbar in a vacuum drying oven for 5 h. Complete solvent removal was monitored by tracking the weight of the coating film during the curing process.

### 2.3. Characterizations

Fourier-transform infrared spectra were registered on a Varian 3100 FTIR spectrometer in the wavenumber range of 400–4000 cm^−1^ with a sample in the form of a thin film cast from solvent (CDCl_3_) on KBr glasses. The ^1^H (400.13 MHz), ^13^C (100.62 MHz), and ^29^Si (79.50 MHz) NMR spectra were obtained on a Bruker DPX-400 spectrometer (Bruker, Bremen, Germany) at 297 K using deuterated chloroform (CDCl_3_) as the solvent with the sample content of 20–30 mg/0.5 mL (sample/CDCl_3_) in 5 mm standard glass NMR tubes. The chemical shifts were expressed in ppm, relative to the solvent resonance signals (7.26 ppm for ^1^H and 77.16 ppm for ^13^C) as the internal standard and tetramethylsilane (0 ppm for ^29^Si) as the external standard. The mass spectrum was obtained using a Shimadzu GCMS-QP5050A mass spectrometer (Shimadzu, Duisburg, Germany) with an injector temperature of 200–250 °C, carrier gas helium, a detector temperature of 290 °C, a quadrupole mass analyzer, and ionization EI (70 eV). Chromatographic separation of trimethoxysilane monomer was carried out on a capillary column SPB-5 (60 m × 0.25 mm × 0.25 μm), with an evaporator temperature of 230 °C, helium carrier gas, a flow rate of 0.7 mL/min, a pressure of 280 kPa, and a gradient from 60 to 250 at 10 °C/min. Ultraviolet–visible spectrum was run on a Shimadzu UV-2450 spectrophotometer (Shimadzu Corporation, Kyoto, Japan). The pH of solutions was measured by using a digital pH electrode connected to a multiparameter laboratory benchtop pH, conductivity, and oxygen meter (HI-2020 edge^®^ Hybrid Multiparameter pH, EC, DO Meter, HANNA Instruments, Leighton Buzzard, UK). The molecular weight and molecular weight distribution of the samples were determined using gel permeation chromatography on a Shimadzu LC-20 Prominence system equipped with a Shimadzu RID-20A differential refractive index detector (Shimadzu Corporation, Kyoto, Japan). The column was 7.5 × 300 mm Agilent PolyPore (PL1113-6500). The temperature was set at 50 °C. The solvent used was *N*,*N*-dimethylformamide, and the flow rate was 1 mL/min. The prepared samples were weighed and then dissolved in DMF at room temperature for 24 h with stirring. The concentration of the solution was 10 mg/mL. Calibration was performed using a series of polystyrene standards, Polystyrene High EasiVials (PL2010-0201), which contained 12 samples with molecular weights ranging from 162 to 6,570,000 g/mol. To determine the hydrodynamic particle diameter (D_h_) of a test sample using dynamic light scattering, a ZetaPALS potential analyzer equipped with a BI-MAS module (Brookhaven Instruments Corporation, Nashua, NH, USA) was used. Deionized water and a 0.1 M NaNO_3_ water–salt solution with a polysiloxane concentration of 0.1 mg/mL were used. The measurements were conducted in a thermostated cuvette at an operating temperature of 25 °C and a scattered light recording angle of 90° at a wavelength of 659 nm. Three sets of measurements of 10 scans each were taken. The results obtained were averaged to give the average diameter of the particles. Thermogravimetric analysis and differential scanning calorimetry were performed using STA 449 Jupiter (Netzsch, Selb, Germany) in an air atmosphere at a heating rate of 5 °C per min from 20 to 800 °C; the weight of the samples was 11 mg. Analysis of the qualitative and quantitative composition of the evolved gaseous thermolysis products was performed using a QMS 403 C Aeolos quadrupole mass spectrometer (Netzsch, Germany) coupled with the thermal analyzer. The D8 ADVANCE Bruker diffractometer (Bruker Corporation, IN, USA), equipped with a CuKα radiation source (wavelength 1.5406 Å) and a scintillation detector, was used to obtain powder X-ray diffraction data. The scans were carried out in the range of diffraction angles 2θ from 5° to 80°, with a step size of 0.02°. The ultraviolet irradiation test was performed using a 400 W high-pressure mercury vapor arc-discharge lamp with a source of ultraviolet radiation in the spectral range of 240–340 nm. Elemental analysis was made on a Thermo Scientific Flash 2000 CHNS-Analyzer (Thermo Fisher Scientific, Cambridge, UK). Gravimetric determination of the silicon content was performed using the method described in the literature [32].

## 3. Results and Discussion

### 3.1. Synthesis and Characterizations of Initial Monomer 1-[3-(Trimethoxysilyl)propyl]piperidine-2,6-dione

**Scheme 1:**
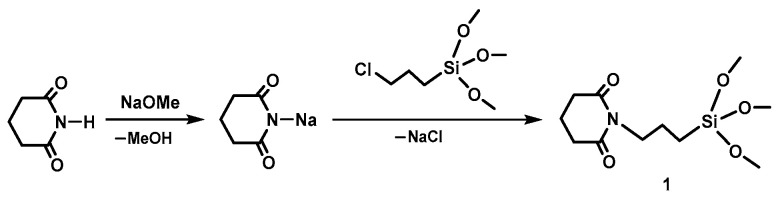
The synthetic route of 1-[3-(trimethoxysilyl)propyl]piperidine-2,6-dione **1**.

Most precursors of polyorganosiloxanes are chlorine-containing silanes. The hydrolysis of organochlorosilanes, as well as the condensation of chlorosilanes and silanols, is accompanied by the elimination of hydrogen chloride gas, which has a significant effect on the process and composition of the resulting products due to the reverse reactions at the Si–O bond and complexation with water [33]. The released toxic hydrogen chloride is a dangerous reagent for the environment and does not comply with the principles of green chemistry, and its disposal presents significant difficulties. Therefore, to obtain silsesquioxanes in an environmentally friendly way due to the potential risks and dangers associated with halogenated chemicals, we used an alternative chlorine-free method for the synthesis of polyorganosiloxanes. Alkoxysilane RSi(OAlk)_n_, a representative of a unique class of organosilicon compounds with hydrolytically active functional groups at the silicon atom, was chosen as the initial monomer. Trifunctional silanes compounds can undergo hydrolytic polycondensation easily, resulting in high molecular weight compounds. The synthesis of alkoxy(alkyl)silane monomers as starting materials is a key process for the environmentally friendly production of polyorganosiloxanes. Such a process can be much more attractive from an economic and environmental point of view compared to chlorine technology. It is known that trimethoxysilanes react much faster compared to triethoxysilyl derivatives under hydrolytic polycondensation conditions [34]. In this regard, in order to search for organoalkoxysilane precursors for the synthesis of new polyorganosilsesquioxanes, we chose the trimethoxysilylpropyl derivative of glutarimide. New 1-[3-(trimethoxysilyl)propyl]piperidine-2,6-dione, a starting monomer for the production of novel polysiloxane materials containing side-chain glutarimide groups, has been synthesized through the nucleophilic substitution reaction of the chlorine atom in (3-chloropropyl)trimethoxysilane on piperidine-2,6-dione group (Scheme 1). Previously, using this method, we successfully synthesized a series of bridged trialkoxysilylalkyl derivatives of nitrogen-containing heterocycles [28,35].

**Figure 1:**
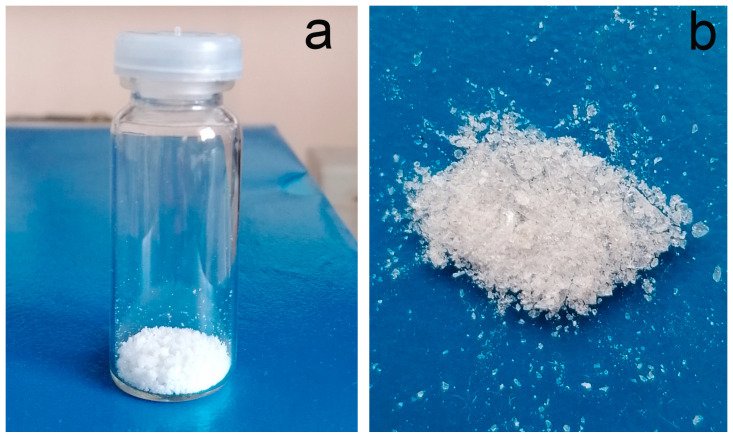
Images of the 1-[3-(trimethoxysilyl)propyl]piperidine-2,6-dione (**a**) and 1-[3-(silsesquioxanyl)propyl]piperidine-2,6-dione **2a** (**b**).

Piperidine-2,6-dione sodium salt, the starting material for the synthesis of the target trimethoxysilylpropyl functionalized imide **1**, is formed as a result of the interaction of sodium methoxide with glutarimide imide at 64 °C for 2 h in a methanol solution. The reaction of the sodium salt with halogenopropyltrimethoxysilane in a stoichiometric molar ratio proceeds by heating in an anhydrous polar solvent *N*,*N*-dimethylformamide in the presence of a dibenzo-24-crown-8-ether catalyst at 70 °C for 2.5 h. During the reaction, the color of the reaction mixture changed from colorless to bright orange. The final colorless crystalline product **1**, isolated by vacuum distillation, is readily soluble in alcohols, CHCl_3_, and polar organic solvents such as DMF, DMSO, and CH_3_CN and easily sublimates under reduced pressure (Figure 1a).

**Figure 2:**
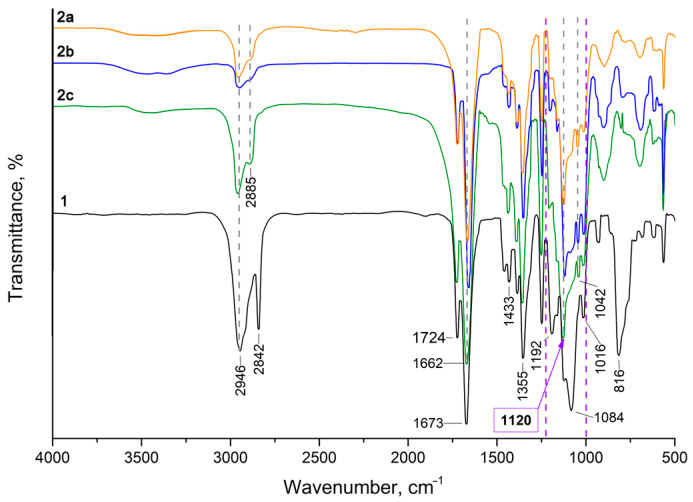
FTIR spectra of 1-[3-(trimethoxysilyl)propyl]piperidine-2,6-dione (**1**) and 1-[3-(silsesquioxanyl)propyl]piperidine-2,6-dione (**2a**–**c**).

The synthesized trimethoxysilane **1** was characterized using elemental analysis, FTIR, ^1^H, ^13^C, ^29^Si NMR spectroscopy, and mass spectrometry. The elemental analysis data are in good agreement with the calculated elemental content. The mass spectrum of trimethoxysilane **1** is characterized by a low-intensity molecular ion with *m*/*z* 275 (0.09%) (Figure S1 in Supplementary Materials). The main peak [Si(OMe)_3_]^+^ with *m*/*z* 121 in the mass spectrum of compound **1** has the highest intensity (100%). The FTIR spectrum of compound **1** shows absorption bands at 2842, 1084, 1016, and 816 cm^−1^ corresponding to methoxysilyl groups (Figure 2). Two characteristic absorption bands at 1724 cm^−1^ and 1673 cm^−1^, respectively, are due to symmetric and asymmetric vibrations of the two C=O groups of the imide ring. This position of the split carbonyl absorption bands is characteristic of many cyclic imides [36,37,38].

**Figure 3:**
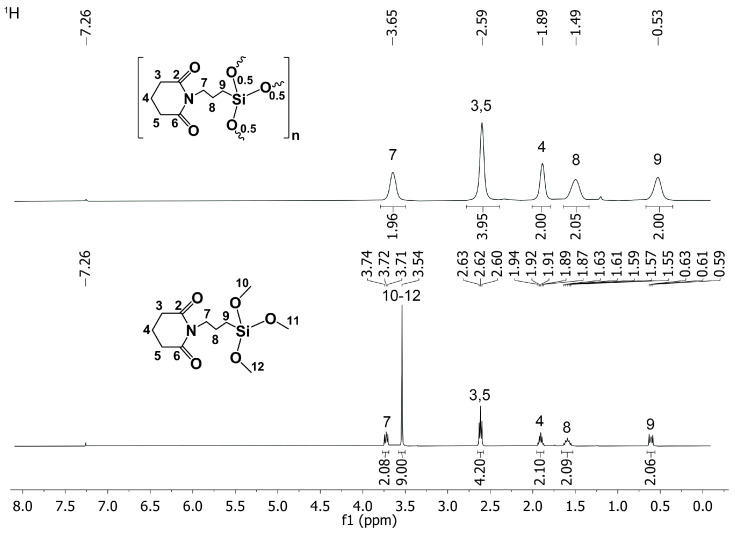
^1^H NMR spectra of 1-[3-(trimethoxysilyl)propyl]piperidine-2,6-dione (down) and 1-[3-(silsesquioxanyl)propyl]piperidine-2,6-dione (**2a**) (up).

The ^1^H NMR spectrum of 1-[3-(trimethoxysilyl)propyl]piperidine-2,6-dione **1** fully corresponds to the declared structure (Figure 3, down). The ^1^H NMR spectrum shows signals of methoxy group protons and glutarimide fragment protons, as well as signals related to the protons of the methylene groups of the propyl bridge. The ratio of integral intensities corresponds to the theoretical one.

**Figure 4:**
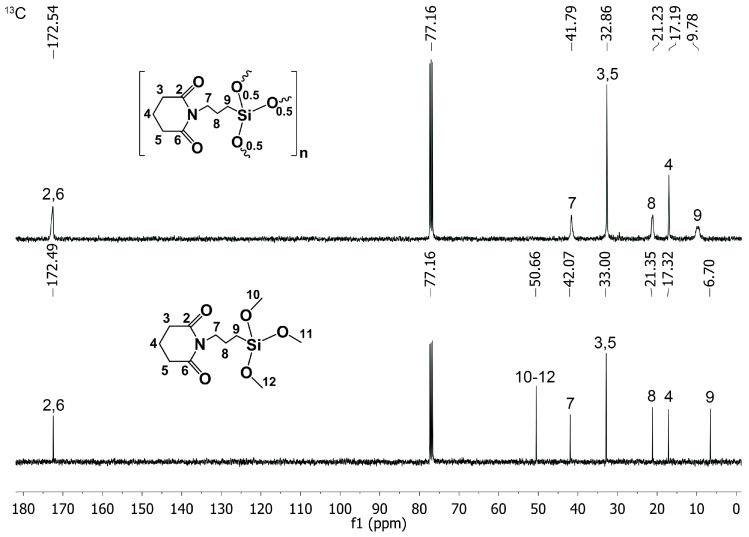
^13^C NMR spectra of 1-[3-(trimethoxysilyl)propyl]piperidine-2,6-dione (down) and 1-[3-(silsesquioxanyl)propyl]piperidine-2,6-dione **2a** (up).

The ^13^C NMR spectrum contains all the signals of carbon atoms of all groups (Figure 4, down), which also confirms the structure of the synthesized silane **1**.

The ^29^Si NMR spectrum of compound **1** contains one signal at δ −42 ppm (Figure S2, in Supplementary Materials), the value of which corresponds to a tetracoordinated silicon atom in trifunctional alkoxysilanes. Thus, both FTIR and ^1^H, ^13^C, and ^29^Si NMR analyses indicated that trimethoxysilane **1** is the target product, which can be successfully used as a precursor for the preparation of silsesquioxanes.

### 3.2. Synthesis and Characterizations of 1-[3-(Silsesquioxanyl)propyl]piperidine-2,6-diones

**Scheme 2:**
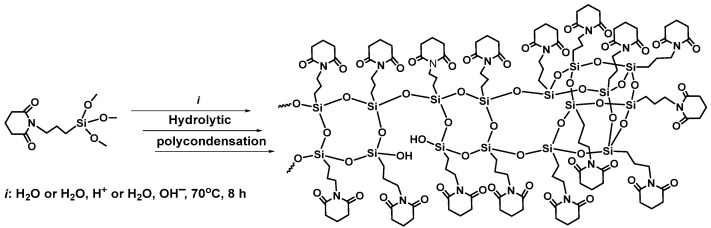
The synthetic route of 1-[3-(silsesquioxanyl)propyl]piperidine-2,6-dione.

A new functional polysilsesquioxanes bearing glutarimide side-chain groups **2a**–**c** were synthesized by the facile reaction of hydrolytic polycondensation of trifunctional 1-[3-(trimethoxysilyl)propyl]piperidine-2,6-dione **1** in an aqueous medium at pH 7 (**2a**), 3 (**2b**), and 10 (**2c**) (Scheme 2).

During hydrolysis, the methoxy groups of trimethoxysilane **1** are hydrolyzed to form silanol and methyl alcohol. Further, the reactive trisilanol enters into a condensation reaction leading to silsesquioxane, the main chain of which consists of a Si–O–Si bond skeleton, in which the silicon atom is bound to the glutarimide fragment by a propylene bridge. Polycondensation occurs in the following two ways: homofunctional condensation, leading to the formation of a siloxane bond and water (condensation of silanol groups), and heterofunctional condensation, leading to the formation of a siloxane bond and alcohol (condensation of silanol and methoxy groups). The reaction was carried out for 8 h at 70 °C. The resulting polysilsesquioxanes are colorless solid products (Figure 1b and Table 1), highly soluble in chloroform, alcohols, acetone, DMF, and DMSO. Based on the data in Table 1, it can be seen that the yields of silsesquioxanes **2a**–**c**, which were synthesized by acid-, base-, or non-catalytic hydrolytic condensations of trimethoxysilane **1**, are similar.

**Figure 5:**
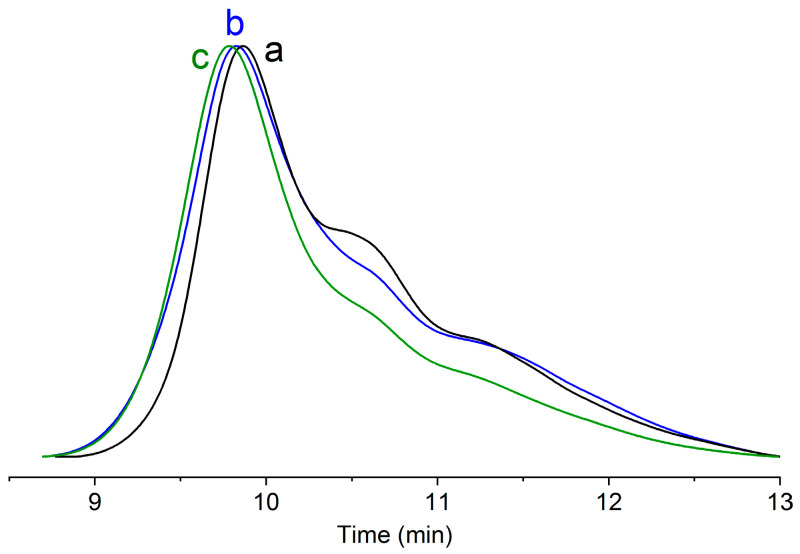
GPC curves of silsesquioxanes **2a**–**c**: a—pH 7, b—pH 3, and c—pH 10.

The molecular weight characteristics of the PSQ-GI **2a**–**c** were determined by gel permeation chromatography using DMF as an eluent. The results obtained are presented in Table 1 and Figure 5. Table 1 shows that polysiloxanes **2a**–**c** synthesized under different conditions of acidity in the reaction medium have similar weight average molecular weights in the range of 10–12 kDa. The PDI (polydispersity index) of these polysiloxanes varies from 1.38 to 1.47, indicating a relatively narrow distribution of molecular weights. The GPC curves indicate the formation, in addition to the main polymer fraction, of a small fraction of lower molecular weight hydrolytic polycondensation products (Figure 5). At the same time, the content of the latter decreases significantly at pH 10. Thus, under base catalysis, the condensation of silanol groups leads to the production of a more narrowly dispersed silsesquioxane with a PDI of 1.38.

The structure of 1-[3-(silsesquioxanyl)propyl]piperidine-2,6-dione **2a**–**c** was characterized by ^1^H, ^13^C, and ^29^Si NMR spectroscopy. The broader signals observed in the ^1^H NMR spectra of the isolated silsesquioxanes **2a**–**c** (Figure 3, up; Figures S3 and S4, in Supplementary Materials) compared to the starting monomer **1** indicate the polymeric nature of the compounds. According to ^1^H NMR spectroscopy data, the signals of methoxy group protons at 3.54 ppm, which are characteristic of trimethoxysilane monomer **1**, are not present in the spectra of the products **2a**–**c** of its hydrolytic polycondensation. This indicates their complete conversion during hydrolysis. The ^13^C NMR spectra of PSQ-GI **2a**–**c** (Figure 4, up; Figures S5 and S6, in Supplementary Materials) show broader signals from the carbon nuclei of the alkyl chain and glutarimide fragments. Signals corresponding to –OCH_3_ groups in the starting 1-[3-(trimethoxysilyl)propyl]piperidine-2,6-dione **1** at 50.65 ppm (Figure 4, down) are not observed in the spectra of the resulting silsesquioxanes.

**Figure 6:**
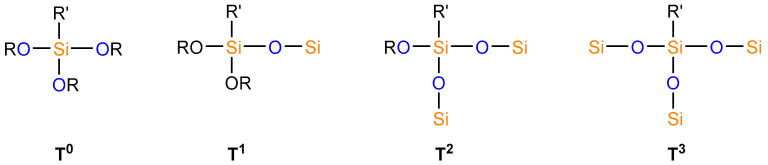
Unit structures for siloxanes and silsesquioxanes.

The degree of condensation of silicon atoms is determined by the number of siloxane bonds. According to the literature [30,39,40], there are four types of silicon atoms: T^0^, T^1^, T^2^, and T^3^, depending on their degree of condensation (Figure 6). The degree of condensation of silicon atoms without siloxane bonds is designated as T^0^. Silicon atoms with one siloxane bond are designated as T^1^, while silicon atoms with two siloxane bonds are designated as T^2^. The designation of T^3^ corresponds to completely condensed silicon atoms, which have three Si–O–Si bonds.

**Figure 7:**
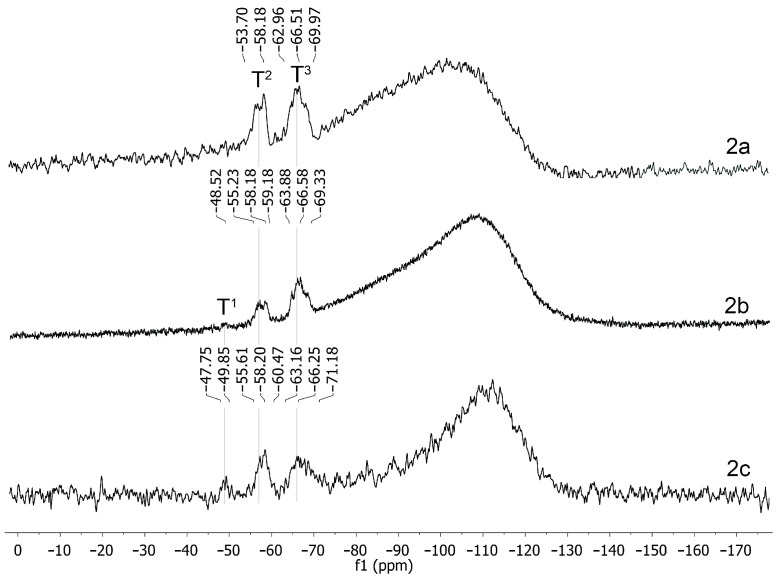
^29^Si NMR spectra of 1-[3-(silsesquioxanyl)propyl]piperidine-2,6-dione (**2a**–**c**).

The ^29^Si NMR spectra of the synthesized silsesquioxanes **2a**–**c** are shown in Figure 7. Each spectrum exhibits chemical shifts in the region from –53 to –60 ppm and from –61 to –71 ppm, corresponding to the structural units T^2^ and T^3^ [31]. The ^29^Si NMR spectra of the polymers **2b** and **2c** show peaks in the range of –46 to –50 ppm that can be assigned to T^1^ species. The chemical shift of the monomer **1** at –42.4 ppm is missing.

The percentage content of structural units T^1^, T^2^, and T^3^, calculated by integrating their individual signals, allows the degree of condensation of polymeric silsesquioxanes to be calculated using Equation (1) [40]:

DC [%] = (3∙T^3^ [%] + 2∙T^2^ [%] + T^1^)/3 (1)

Using the degree of condensation (DC), the residual amount of hydroxyl groups in silsesquioxane can be calculated by applying Formula (2) [39]:

OH [%] = 100% − DC [%](2)

The obtained values are presented in Table 2.

Calculation of the ratio of the integral signal intensities of silicon atoms in different structures, taking into account their relative contribution to the polycondensation products, showed the content of silanol groups in the range of 13–15%.

This indicates significant incompleteness of the second stage of hydrolytic polycondensation, namely, silanol condensation. From Table 2, it can be seen that silsesquioxanes **2a**–**c**, obtained at different pH values, are characterized by a fairly high degree of condensation of about 87%.

The value of the width at half-height (w_1/2_) of the resonance peak of the silicon atom of T^3^ SQ units is an identifier of the ladder structure of polysilsesquioxane; the narrower w_1/2_, the higher the structure regularity. The values of half-peak (w_1/2_) 4.6 (**2a**), 4.4 (**2b**), and 4.9 ppm (**2c**) indicate the presence of structural defects in the silsesquioxanes **2a**–**c** backbone, respectively.

The chemical structure of polysilsesquioxanes **2a**–**c** obtained by hydrolysis and condensation of precursor 1 in water at pH 7 or in acidic or alkaline medium in the presence of HCl (pH 3) or NaOH (pH 10) was characterized by Fourier-transform infrared spectroscopy (Figure 2). In general, the spectra of compounds **2a**–**c** are almost identical. The absorption bands at 1192 and 816 cm^−1^ corresponding to the methoxysilyl groups of the initial monomer **1** disappeared, confirming the completion of the hydrolysis reaction [41]. The broad absorption band at 3376 cm^−1^ was stretching vibrations of residual associated Si–OH groups. During hydrolysis and condensation, the glutarimide group remains unaffected. This is evidenced by absorption bands at 1722 cm^−1^ (ν_s_ C=O) and 1662 cm^−1^ (ν_as_ C=O) and 1460–1435 cm^−1^ and 1387–1353 cm^−1^ (C–C bond deformation vibrations of the glutarimide ring). Compared to the FTIR spectrum of 1-[3-(trimethoxysilyl)propyl]-piperidine-2,6-dione, the absorption peaks of the C=O group at 1724 and 1673 cm^−1^ are shifted to the low-frequency region by 2 and 11 cm^−1^ in samples **2a**–**c**.

These results represent a different arrangement of carbonyl groups in the PSQ-GI molecule. The change in the character of the absorption bands in the 1200–1000 cm^−1^ region indicates that the polycondensation reaction is taking place. This broad complex Si–O–Si valence absorption band demonstrates the presence of various silsesquioxane species. The new peak at 1042 cm^−1^ (ν_ring-sym_), which appears in the IR spectrum of silsesquioxanes **2a**–**c** (Figure 2), can be attributed to the stretching vibrations of the siloxane bonds in low-symmetry structures such as ladder-like, open cage, or random network. The observed high intensity of the absorption peak of the Si–O–Si bond at 1120 cm^−1^ suggests the formation of a specific structure in PSQ-GI, which is likely to be a cage [42,43]. Thus, the IR spectra showed that the resulting polymer molecules **2a**–**c** probably have a mixed ladder-like and cage structure.

**Figure 8:**
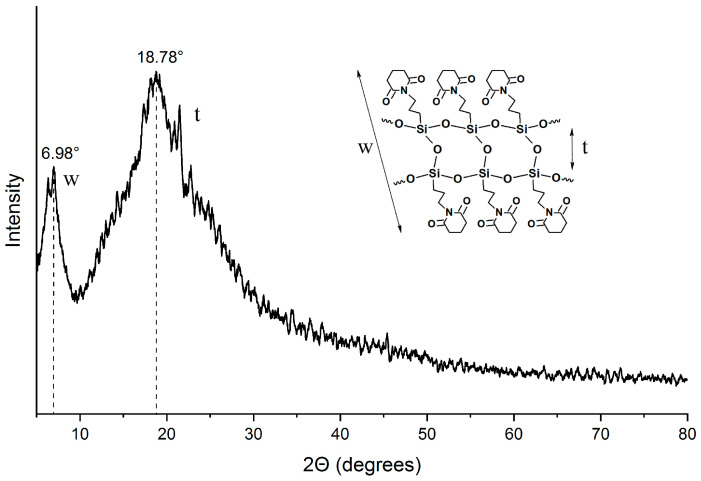
XRD pattern of polysilsesquioxane **2a**.

The powder X-ray diffraction method was used to study the 1-[3-(silsesquioxanyl)propyl]piperidine-2,6-dione **2a** structure. The X-ray diffraction pattern of silsesquioxane **2a** demonstrates two distinct characteristic diffraction halos, which is in good agreement with the literature data regarding the structure analysis of the ladder polymers by XRD (Figure 8) [44,45,46,47,48]. The first halo (w) at 2θ = 6.98° indicates the intramolecular chain-to-chain distance, corresponding to the width of a double-chained molecule of ladder organosilicon polymers. The 1-[3-(silsesquioxanyl)propyl]piperidine-2,6-dione **2a** is amorphous, but the narrow and sharp diffraction peak (w) with high intensity indicates that the polymer has a relatively regular ladder-like skeleton, the rigidity of which limits movement around the longitudinal axis. The second diffuse halo (t), covering a wide range of diffraction angles with a maximum at 2θ 18.78°, corresponds to the average thickness of the ladder-like polymer chain. The average thickness of the macromolecular chain of polysilsesquioxane **2a** was found to be at 4.72 Å, and the distance between two glutarimide side-chain groups through the siloxane backbone of the ladder structure was 12.65 Å.

The effective hydrodynamic diameters of polymer coils of synthesized 1-[3-(silsesquioxanyl)propyl]piperidine-2,6-dione **2a** were measured by dynamic light scattering in water and aqueous salt solution. The hydrodynamic particle diameter was calculated using the Stokes–Einstein relation (Equation (3)), according to which the diffusion velocity is inversely proportional to the particle size [49]:

D = k_B_T/3πηD_h_ (3)

where k_B_ is the Boltzmann constant (1.380 × 10^−23^ kg·m^2^·s^−2^·K^−1^), T (K) is an absolute temperature, η (kg·m^−1^·s^−1^) is the viscosity of medium, D_h_ (m) is the hydrodynamic particle diameter, and D (m^2^·s^−1^) is the diffusion coefficient.

**Figure 9:**
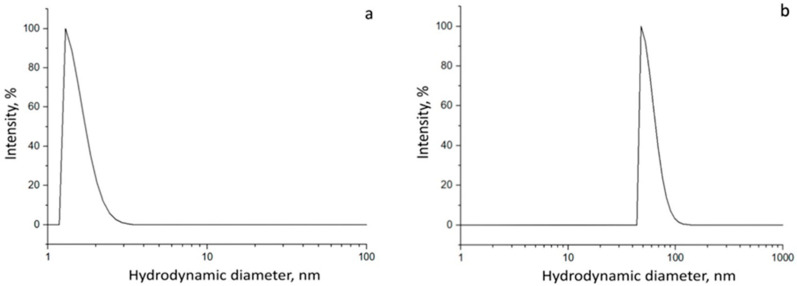
Distribution of effective diameters of scattering particles of PSQ-GI **2a**: (**a**) in aqueous salt solution and (**b**) in water.

The obtained histograms of the dependence of the signal intensity on the hydrodynamic diameter in aqueous and water-salt media indicate that silsesquioxane **2a** forms a colloidal system with a monomodal particle size distribution (Figure 9).

The sizes of scattering particles of silsesquioxane **2a**, corresponding to the effective hydrodynamic diameter, differ in aqueous and aqueous salt media. In an aqueous salt solution, the diameter of the scattering particles is 1.3 nm (Figure 9a). Traces of a low-molecular-weight fraction are present in an aqueous solution of silsesquioxane **2a**, but most of the polysiloxane is in an associated state. The average hydrodynamic diameter of the particles is 46 nm (Figure 9b). Macromolecules of PSQ-GI **2a** in water are in an associated state, forming supramolecular structures due to the intermolecular interaction of individual macromolecules.

**Figure 10:**
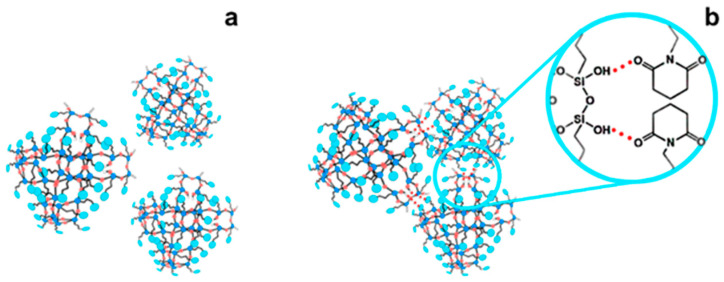
1-[3-(Silsesquioxanyl)propyl]piperidine-2,6-dione **2a**: (**a**) in aqueous salt solution (unassociated state) and (**b**) in water (associated state).

The formation of these associated structures can be due to hydrogen bonding between carbonyl oxygen atoms in glutarimide fragments and hydroxyl groups Si–OH in different macromolecular siloxane chains (Figure 10).

**Figure 11:**
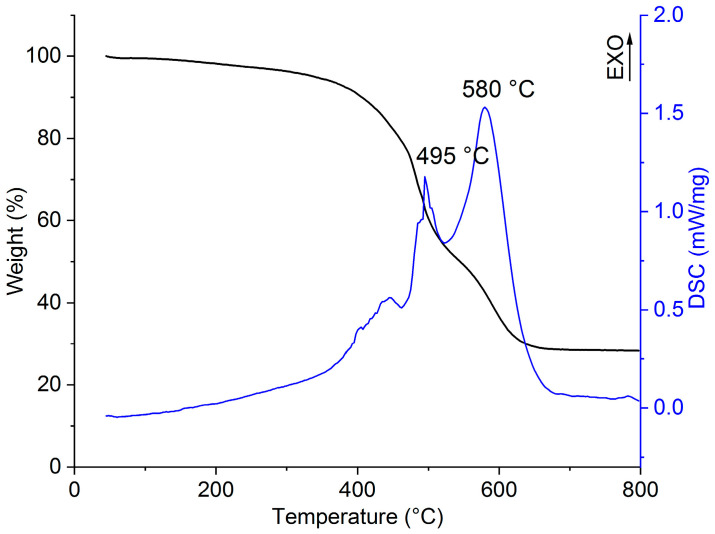
TGA (black line) and DSC (blue line) curves of PSQ-GI **2a** (under air, 5 °C/min).

**Figure 12:**
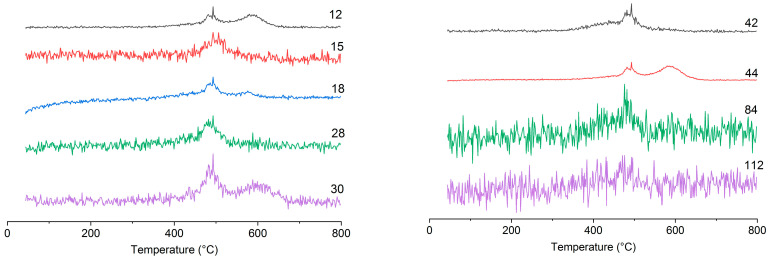
Mass spectra of PSQ-GI **2a** under air.

The polysilsesquioxane **2a** thermal properties were characterized by thermogravimetric analysis and differential scanning calorimetry in an oxidative atmosphere, the results of which are given in Figure 11. TGA data show that silsesquioxane **2a** is stable up to 335 °C (T_d5_), after which weight loss started, associated with thermal oxidative degradation. The pyrolysis temperature of PSQ-GI **2a** with a weight loss of 10% was 405 °C. This is due to the presence of thermally stable glutarimide side-chain groups and the Si–O–Si siloxane bond framework. The initial weight loss of the sample can be explained by the removal of water. The unreacted OH groups present in product **2a** condense, resulting in an increase in the degree of cross-linking. Weight loss associated with thermal oxidative destruction is characterized by two main stages. The first degradation stage with a 37% weight loss in the range from 405 °C to 524 °C is attributed to the thermal decomposition of the organic side chain groups of the silsesquioxane. In this case, an exothermic peak with a maximum at 495 °C is observed on the DSC curve. The mass loss is accompanied by peaks in the ion current curves with mass numbers corresponding to low-molecular-weight products of thermal oxidative degradation with *m*/*z* 12 (C), 28 (CO), 30 (NO, CH_2_O), 42 (NCO), and 44 (CO_2_) (Figure 12).

Products with high *m*/*z* 80, 81, 84, 111, 112, and 113 in the mass spectrum are also identified, which probably corresponds to the cleavage of the N–C bond and elimination of the glutarimide fragment. The second degradation stage in the temperature range of 540–660 °C with a maximum at 580 °C and with 21% weight loss was caused by the pyrolytic decomposition of glutarimide groups, oxidation of free carbon, and thermal degradation of the Si–O–Si main chain. Thermal degradation of the Si–O–Si backbone was mainly caused by a random cleavage reaction in which an oxygen atom recombined with a non-adjacent silicon atom within the same backbone to form volatile cyclic oligomers [50]. The mass loss is accompanied by peaks in the ion current curves with *m*/*z* 12 (C), 30 (C_2_H_6_), and 44 (CO_2_) (Figure 12). The residual mass of 28% can be attributed to SiO_2_ and char formed during complete decomposition of the polymer.

### 3.3. A Polymer Film Coating PSQ-GI

**Figure 13:**
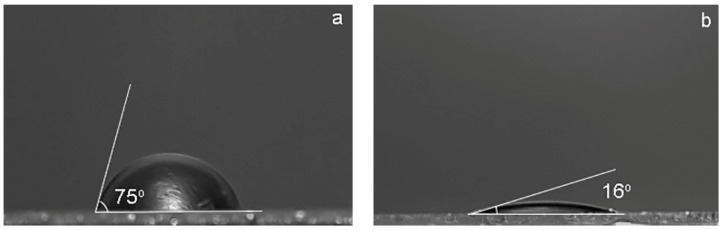
Photograph of a water droplet: (**a**) deposited on the surface of PSQ-GI polymer film coating on silicate glass and (**b**) deposited on untreated silicate glass.

A polymer film coating based on silsesquioxane **2a** was obtained and deposited on a flat glass substrate using the drop-casting method. Contact angle analysis was used to evaluate its surface properties. Figure 13a shows a photograph of a water droplet (2 μL) deposited on the surface of a PSQ-GI polymer film coating on silicate glass. The contact angle of water was 75°. This value is greater than that for deposited on untreated silicate glass (Figure 13b), but less than 90°, that is, the obtained PSQ-GI polymer film coating belongs to hydrophilic materials.

**Figure 14:**
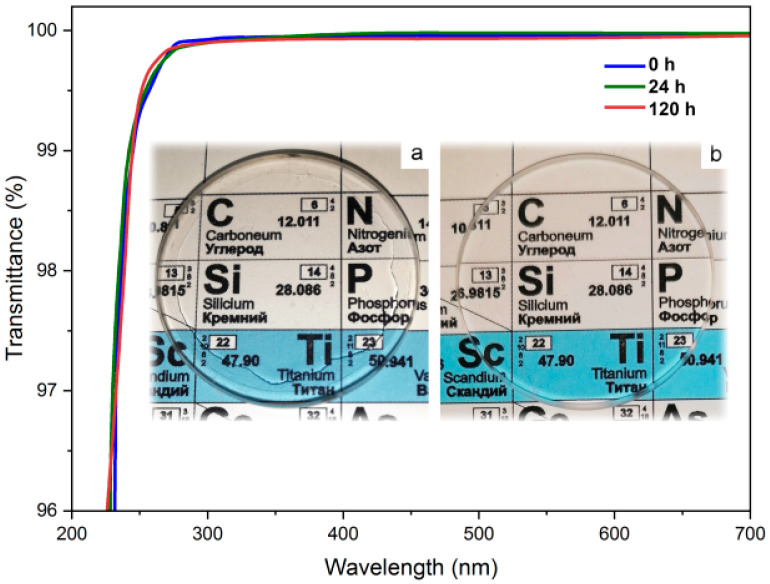
UV-Vis spectrum and photographs (inset) of a polymer-coated glass substrate (**a**) and an uncoated glass substrate (**b**).

The resulting polymer film coating was colorless and transparent, as shown in Figure 14.

The transmittance of the polymer film coating was measured by UV-Vis spectroscopy. The transmission spectra of the polymer film coating after different irradiation times were used to investigate its resistance to UV radiation (Figure 14). The initial transmission spectrum of the film coating showed a high transmission (98%) in the visible wavelength range and in the near ultraviolet region of the spectrum. In the ultraviolet region of the spectrum below 277 nm, the transmittance was reduced due to the absorption of UV radiation by the chromophoric carbonyl groups of the glutarimide moieties of silsesquioxane **2a**. During UV irradiation for 24 h, there were no significant changes in the transmittance of the film coating. This is probably due to the fact that glutarimide fragments act as a UV-absorbing material that protects against ultraviolet radiation, effectively scattering and absorbing light in the wavelength range below 300 nm, and ultraviolet rays practically do not penetrate through the polymer coating film.

When the polymer film is exposed to UV irradiation for a longer time (120 h), the ultraviolet/visible spectrum remains almost unchanged, indicating its resistance to UV radiation. The absence of weight loss of the film coating depending on the time of exposure to UV radiation also indicates its resistance to UV radiation.

Thus, using the UV spectroscopy method, which allows studying solid polymer films, it was shown that the polymer film coating based on silsesquioxane **2a**, containing chromophore groups C=O, can be used as a promising polymer material for protection against UV radiation.

## 4. Conclusions

In summary, this study demonstrates a simple and universal approach to the formation of novel functional thermally stable soluble polysilsesquioxane bearing glutarimide side-chain groups, which lays the foundation for the synthesis of polysiloxanes with various functionality. A previously unknown trifunctional (trimethoxysilylpropyl)glutarimide allowed the use of an environmentally friendly (chlorine-free) approach to create a functional polyorganosilsesquioxane. Hydrolytic polycondensation in water or in an acidic or alkaline medium provides the production of silsesquioxanes with a weight average molecular weight of 10–12 kDa and a low polydispersity index of 1.38–1.47, which indicates the formation of silsesquioxanes with a uniform distribution according to the degree of polycondensation. A totality of research methods (IR, NMR, and XRD) indicate that the synthesized silsesquioxanes have a mixed ladder and cage structure with defects in the form of incompletely condensed silanol groups. Incompletely condensed silsesquioxanes with residual silanol groups are of interest due to the possibility of their use in curable composition modification, as well as for emulsions that impart surface hydrophobicity and for producing optically transparent composite films. The powder X-ray diffraction method showed the amorphous nature of the obtained silsesquioxane. According to DLS, the imide fragments of silsesquioxane provide its solvation in an aqueous medium, including the formation of supramolecular structures due to intermacromolecular association with non-condensed hydroxyl groups. The resulting polysilsesquioxane has relatively high thermal stability (T_d5_ = 335 °C and T_d10_ = 405 °C). Polymer film coatings based on silsesquioxane with glutarimide side-chain fragments containing chromophoric carbonyl groups are characterized by 98% transmittance in the visible spectral region and demonstrate resistance to ultraviolet radiation for a long time. These properties make them promising polymeric materials for UV protection.

## Figures and Tables

**Table 1:** Characteristics of silsesquioxanes **2a**–**c**.

Silsesquioxane		pH	Yield, %	M_n_, kDa	M_w_, kDa	Đ
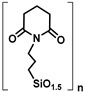	**2a**	7	87	7.1	10.1	1.42
	**2b**	3	78	7.3	10.7	1.47
	**2c**	10	79	8.5	11.7	1.38

**Table 2:** The ratio of condensation products T^1^, T^2^, and T^3^, the degree of condensation, and the proportion of residual Si–OH groups.

Compound	pH	T^1^ (%)	T^2^ (%)	T^3^ (%)	DC (%)	OH (%)
**2a**	7.0	–	40.83	59.17	86.39	13.61
**2b**	3.0	6.79	31.48	61.73	84.98	15.02
**2c**	10.0	2.53	34.18	63.29	86.92	13.08

## Data Availability

The original contributions presented in this study are included in the article/Supplementary Materials. Further inquiries can be directed to the corresponding author.

## Supplementary Materials

The following supporting information can be downloaded at: https://www.mdpi.com/article/10.3390/polym16233235/s1, ^1^H, ^13^C, and ^29^Si NMR spectra of compounds **1**, **2b**, and **2c**.
