# Supplementary material for: Synthesis, Characterization, and Application Prospects of Novel Soluble Polysilsesquioxane Bearing Glutarimide Side-Chain Groups
Source: Polymers (Basel). 2024 Nov 21;16(23):3235. doi: 10.3390/polym16233235 (PMC11644388; doi:10.3390/polym16233235)
Supplement: Supplementary file 1 [file polymers-16-03235-s001.zip › polymers-3321433-supplementary.pdf]

Synthesis, characterization and application prospects of novel soluble polysilsesquioxane bearing glutarimide side-chain groups

Yuliya Bolgova, Artem Emel'yanov, Olga Trofimova, Anastasiya Ivanova, Alexander Albanov, Nadezhda Kuznetsova, Tatyana Semenova, Alexander Pozdnyakov

A.E. Favorsky Irkutsk Institute of Chemistry of the Siberian Branch of the Russian Academy of Sciences, 1 Favorsky Str., Irkutsk, 664033, Russian Federation

Table of Contents:

Fig. S1. MS spectrum of compound 1 S2

Fig. S2. ^{29}Si NMR spectrum of compound 1 in $CDCl_3$ S3

Fig. S3. ^{1}H NMR spectrum of compounds 2b in $CDCl_3$ S4

Fig. S4. ^{1}H NMR spectrum of compounds 2c in $CDCl_3$ S4

Fig. S5. ^{13}C NMR spectrum of compounds 2b in $CDCl_3$ S5

Fig. S6. ^{13}C NMR spectrum of compounds 2c in $CDCl_3$ S5

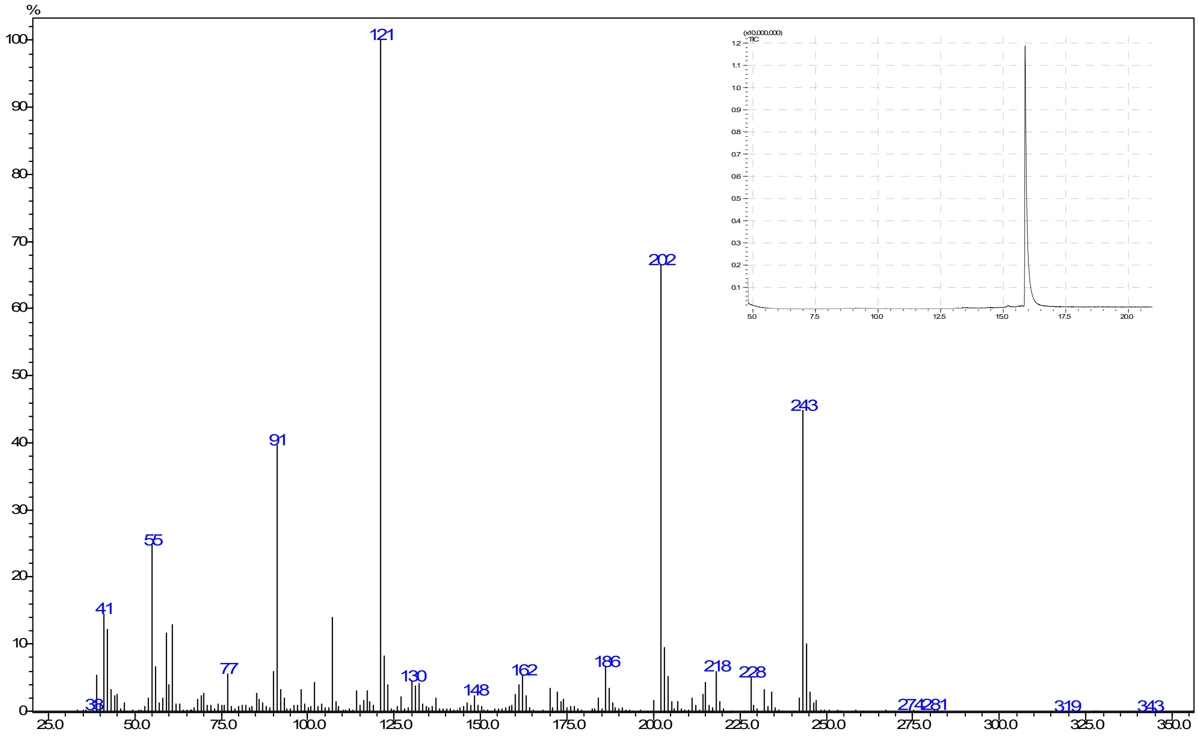

Fig. S1. MS spectrum of 1-[3-(trimethoxysilyl)propyl]piperidine-2,6-dione (1).

Mass Table

m/z	Abs. Inten.	Rel. Inten. (%)
275.30	1727	0.09
274.30	1985	0.11
121.10	1868571	100.00
202.10	1242311	66.48
243.15	839123	44.91
91.10	741797	39.70
61.00	242601	12.98
59.00	219466	11.75
244.15	187576	10.04
90.10	110115	5.89
77.05	102895	5.51
162.15	100079	5.36

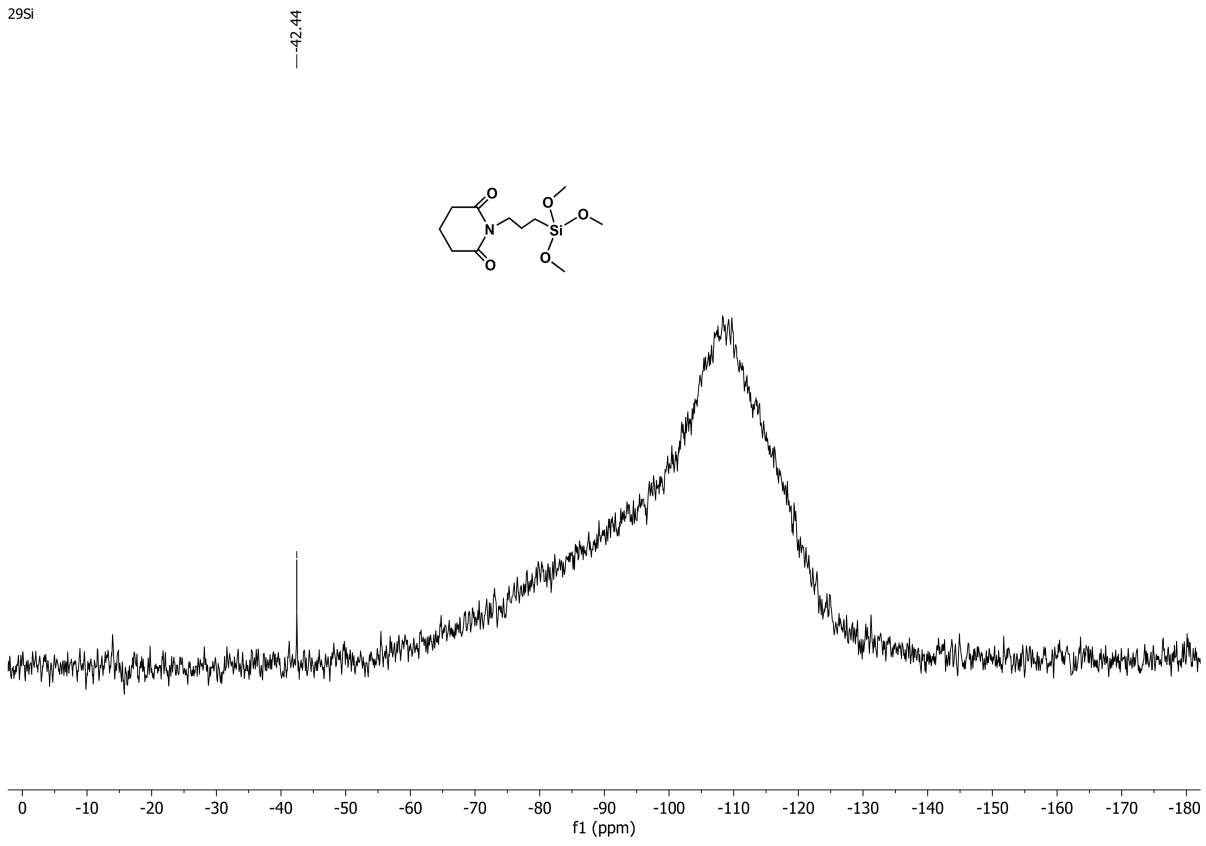

Fig. S2. ^{29}Si NMR spectrum of 1-[3-(trimethoxysilyl)propyl]piperidine-2,6-dione (1) in $CDCl_3$.

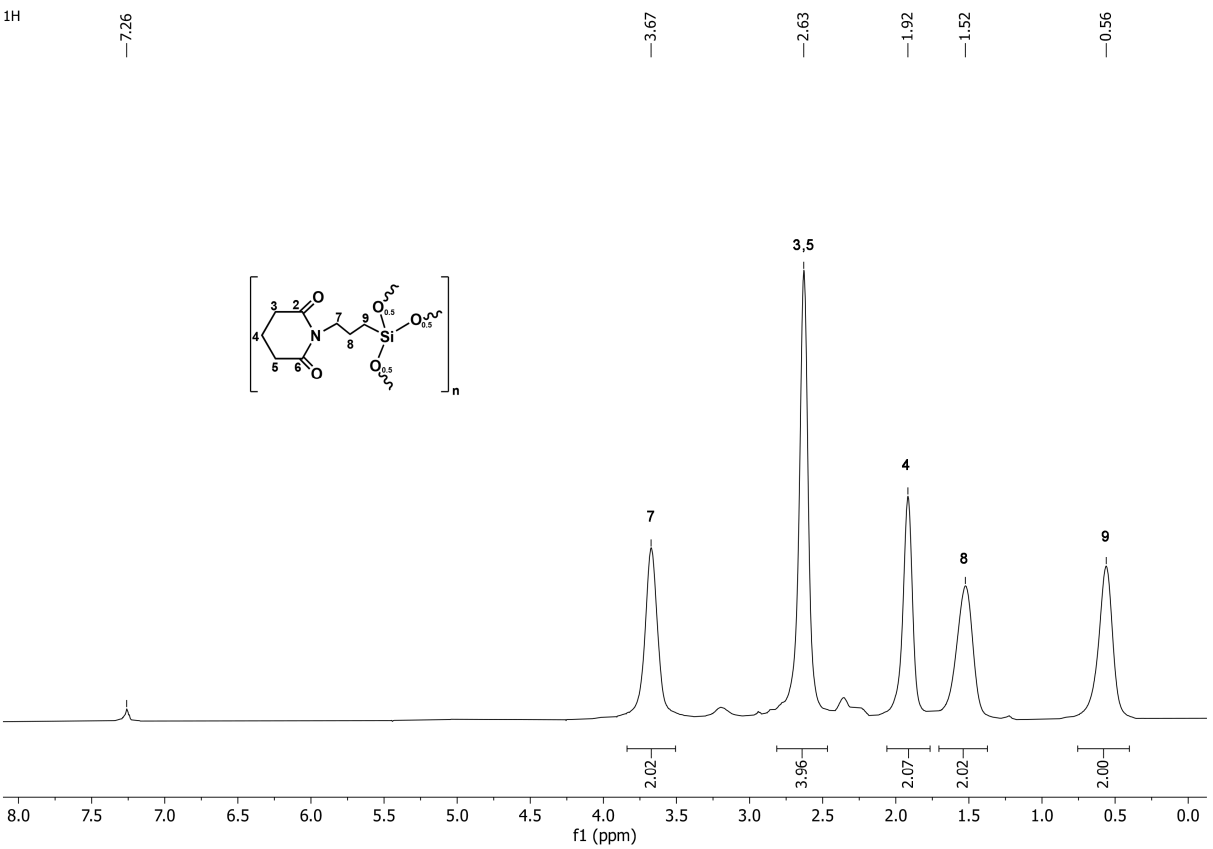

Fig. S3. ^{1}H NMR spectrum of 1-[3-(silsesquioxanyl)propyl]piperidine-2,6-dione (2b) in $CDCl_3$.

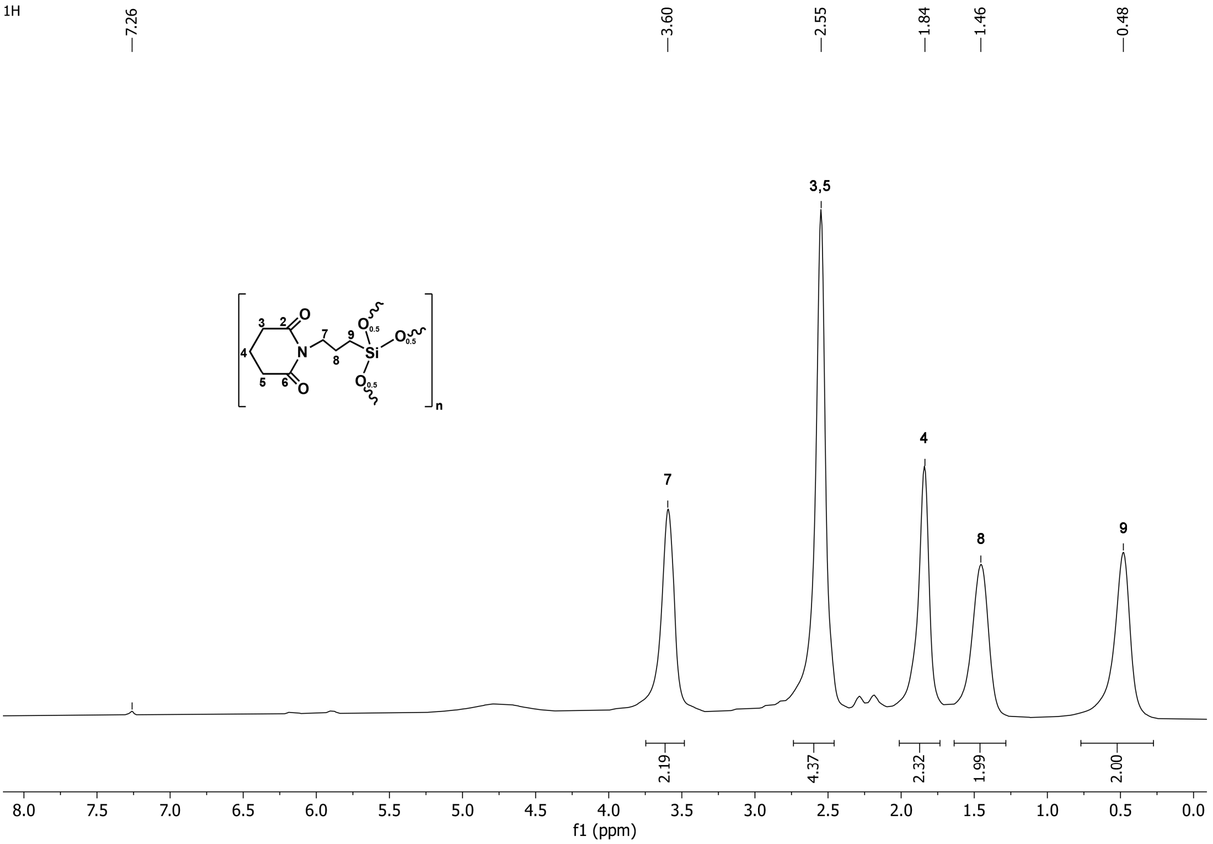

Fig. S4. ^{1}H NMR spectrum of 1-[3-(silsesquioxanyl)propyl]piperidine-2,6-dione (2c) in $CDCl_3$.

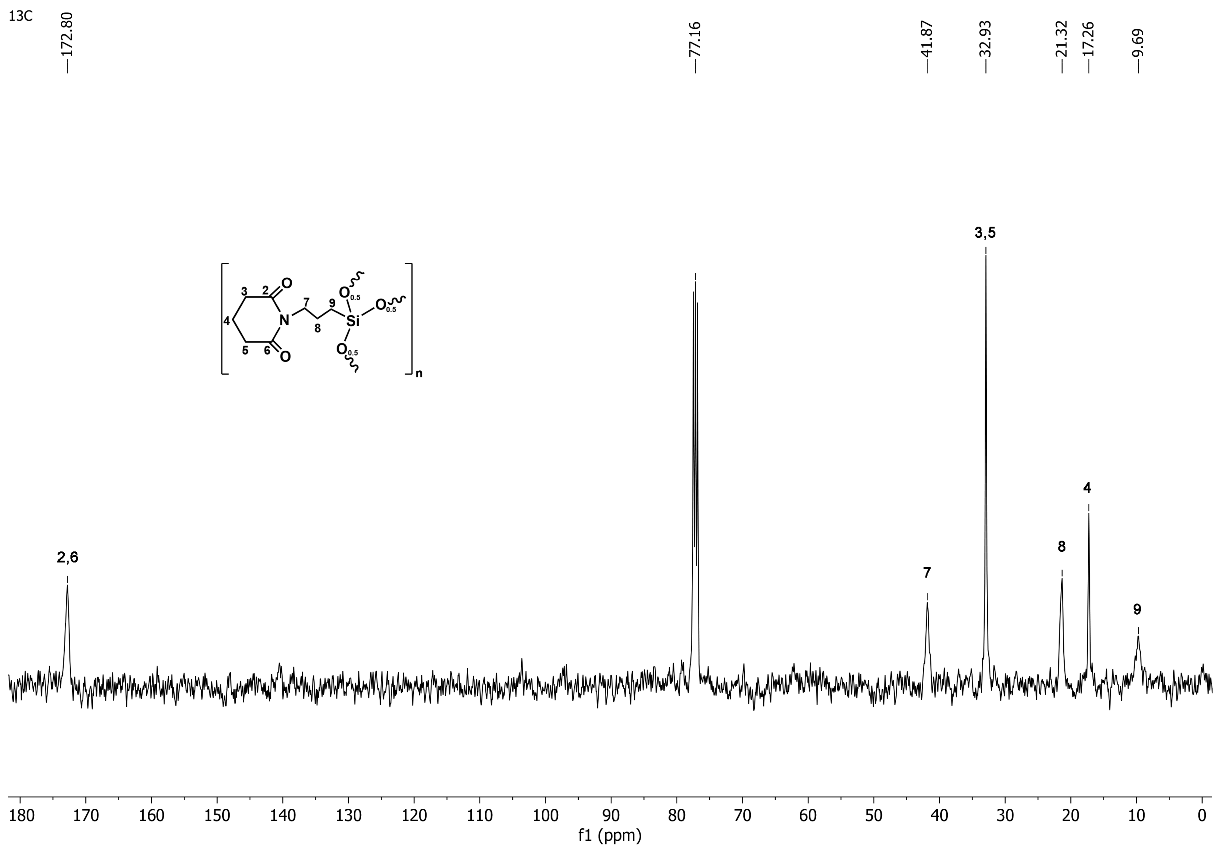

Fig. S5. ^{13}C NMR spectrum of 1-[3-(silsesquioxanyl)propyl]piperidine-2,6-dione (2b) in $CDCl_3$.

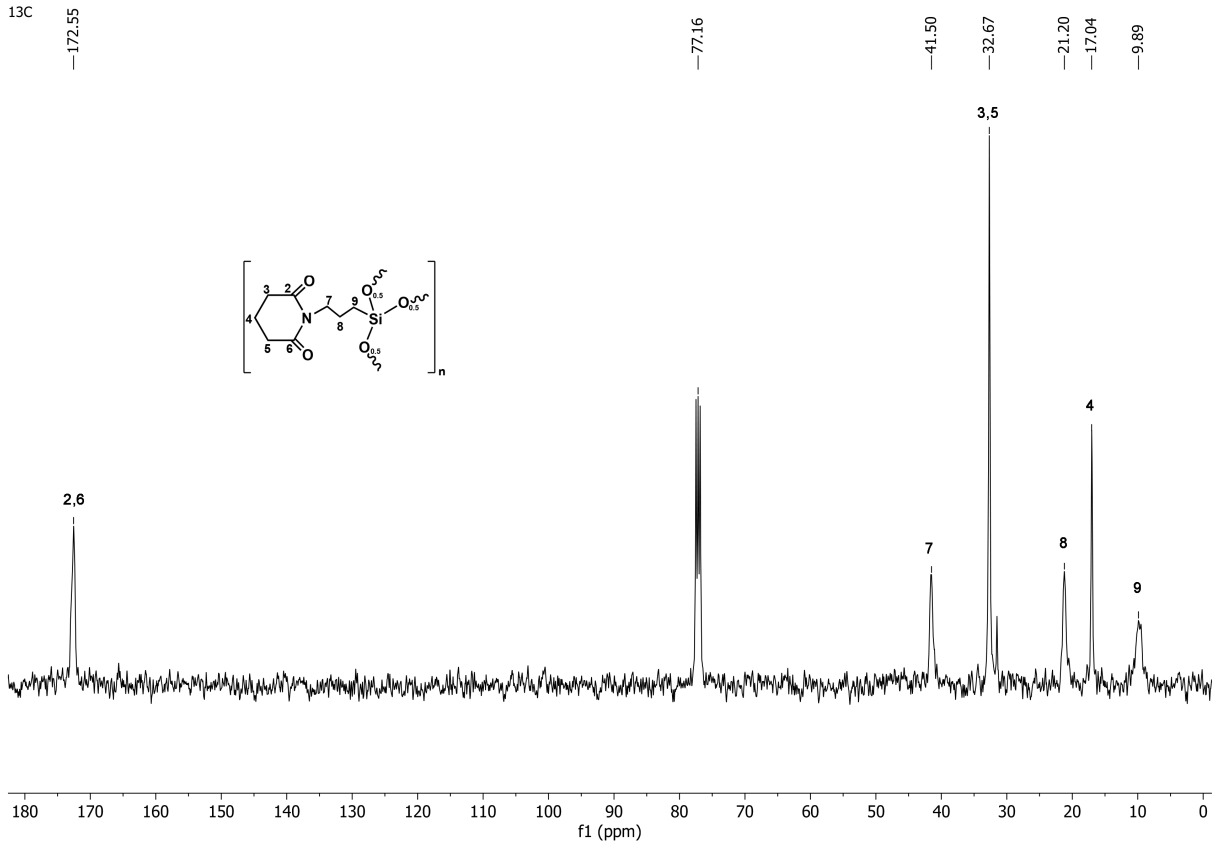

Fig. S6. ^{13}C NMR spectrum of 1-[3-(silsesquioxanyl)propyl]piperidine-2,6-dione (2c) in $CDCl_3$.
